# Supplementary material for: Bibliometric Analysis and Visualization Mapping of Anthrax Vaccine Publications from 1991 through 2021
Source: Vaccines (Basel). 2022 Jun 23;10(7):1007. doi: 10.3390/vaccines10071007 (PMC9316950; doi:10.3390/vaccines10071007)
Supplement: Supplementary file 1 [file vaccines-10-01007-s001.zip › vaccines-1710653-supplementary.pdf]

Supplementary Materials

Supplementary Table S1. All countries or regions involved in anthrax vaccine research between 1991 and 2021.

	Country	Articles	Percentage	Total Global Citations
1	USA	663	60.8	22200
2	UK	97	8.9	3052
3	India	64	5.9	1070
4	China	55	5.0	737
5	Germany	41	3.8	1044
6	Israel	41	3.8	1807
7	France	36	3.3	1366
8	Unknown	34	3.1	531
9	Canada	27	2.5	687
10	Italy	24	2.2	498
11	South Korea	23	2.1	265
12	Iran	17	1.6	74
13	Russia	17	1.6	254
14	Australia	14	1.3	168
15	Japan	14	1.3	325
16	South Africa	12	1.1	217
17	Turkey	11	1.0	129
18	Switzerland	10	0.9	432
19	Brazil	7	0.6	265
20	Taiwan	6	0.6	196
21	Egypt	5	0.5	31
22	Sweden	5	0.5	121
23	Argentina	4	0.4	50
24	Belgium	4	0.4	280
25	Mexico	4	0.4	18
26	Netherlands	4	0.4	145
27	Nigeria	4	0.4	21
28	Poland	4	0.4	35
29	Zambia	4	0.4	21
30	Bangladesh	3	0.3	30
31	Colombia	3	0.3	80
32	Denmark	3	0.3	58
33	Ethiopia	3	0.3	17
34	Spain	3	0.3	90
35	Uganda	3	0.3	34
36	Armenia	2	0.2	11
37	Austria	2	0.2	11
38	Chad	2	0.2	31
39	Cote Ivoire	2	0.2	10
40	Czech Republic	2	0.2	57
41	Georgia	2	0.2	23
42	Indonesia	2	0.2	14
43	Mongolia	2	0.2	8
44	New Zealand	2	0.2	28
45	Norway	2	0.2	656
46	Portugal	2	0.2	74

47	Sudan	2	0.2	1
48	Chile	1	0.1	52
49	Croatia	1	0.1	4
50	Finland	1	0.1	13
51	Ghana	1	0.1	23
52	Greece	1	0.1	35
53	Ireland	1	0.1	25
54	Jordan	1	0.1	10
55	Kazakhstan	1	0.1	55
56	Malaysia	1	0.1	48
57	Mozambique	1	0.1	2
58	Saudi Arabia	1	0.1	17
59	Senegal	1	0.1	19
60	Tanzania	1	0.1	4
61	Thailand	1	0.1	2
62	United Arab Emirates	1	0.1	1
63	Vietnam	1	0.1	70
64	Zimbabwe	1	0.1	2
